# Characterization of Jasmonoyl-Isoleucine (JA-Ile) Hormonal Catabolic Pathways in Rice upon Wounding and Salt Stress

**DOI:** 10.1186/s12284-019-0303-0

**Published:** 2019-06-25

**Authors:** Mohamed Hazman, Martin Sühnel, Sandra Schäfer, Julie Zumsteg, Agnès Lesot, Fréderic Beltran, Valentin Marquis, Laurence Herrgott, Laurence Miesch, Michael Riemann, Thierry Heitz

**Affiliations:** 1 Institut de Biologie Moléculaire des Plantes (IBMP) du CNRS, Université de Strasbourg, Strasbourg, France; 2 Agricultural Genetic Engineering Research Institute (AGERI), Agricultural Research Centre (ARC), Giza, 12619 Egypt; 3 Karlsruhe Institute of Technology, Botanical Institute, Karlsruhe, Germany; 4 Synthèse Organique et Phytochimie (SOPhy), Institut de Chimie, Université de Strasbourg, CNRS, Strasbourg, France

**Keywords:** Jasmonate catabolism, Wounding, Salt stress, JA-Ile, CYP94, Amidohydrolase

## Abstract

**Background:**

Jasmonate (JA) signaling and functions have been established in rice development and response to a range of biotic or abiotic stress conditions. However, information on the molecular actors and mechanisms underlying turnover of the bioactive jasmonoyl-isoleucine (JA-Ile) is very limited in this plant species.

**Results:**

Here we explored two gene families in rice in which some members were described previously in Arabidopsis to encode enzymes metabolizing JA-Ile hormone, namely cytochrome P450 of the CYP94 subfamily (CYP94, 20 members) and amidohydrolases (AH, 9 members). The CYP94D subclade, of unknown function, was most represented in the rice genome with about 10 genes. We used phylogeny and gene expression analysis to narrow the study to candidate members that could mediate JA-Ile catabolism upon leaf wounding used as mimic of insect chewing or seedling exposure to salt, two stresses triggering jasmonate metabolism and signaling. Both treatments induced specific transcriptional changes, along with accumulation of JA-Ile and a complex array of oxidized jasmonate catabolites, with some of these responses being abolished in the *JASMONATE RESISTANT 1* (*jar1*) mutant. However, upon response to salt, a lower dependence on JAR1 was evidenced. Dynamics of *CYP94B5*, *CYP94C2*, *CYP94C4* and *AH7* transcripts matched best the accumulation of JA-Ile catabolites. To gain direct insight into JA-Ile metabolizing activities, recombinant expression of some selected genes was undertaken in yeast and bacteria. CYP94B5 was demonstrated to catalyze C12-hydroxylation of JA-Ile, whereas similarly to its Arabidopsis bi-functional homolog IAR3, AH8 performed cleavage of JA-Ile and auxin-alanine conjugates.

**Conclusions:**

Our data shed light on two rice gene families encoding enzymes related to hormone homeostasis. Expression data along with JA profiling and functional analysis identifies likely actors of JA-Ile catabolism in rice seedlings. This knowledge will now enable to better understand the metabolic fate of JA-Ile and engineer optimized JA signaling under stress conditions.

**Electronic supplementary material:**

The online version of this article (10.1186/s12284-019-0303-0) contains supplementary material, which is available to authorized users.

## Background

Jasmonate hormones (JAs) play major roles in plant development and their responses to environmental challenges. Essential functions of this hormonal pathway have been demonstrated in vegetative or reproductive processes, and adaptation to an array of adverse conditions, particularly in Arabidopsis and other dicotyledonous plants (Wasternack and Hause 2013). Through complex interplays with other phytohormones, JAs control a wide spectrum of induced responses, including the accumulation of defense proteins, or enzymes directing the formation of specialized metabolites, both increasing survival capacity against herbivorous insects or necrotrophic microbial pathogens (Campos et al. 2014). In addition, JA signaling also impacts tolerance to different types of abiotic stress (Kazan 2015), making this hormone a critical hub for translating environmental cues into relevant physiological adaptations.

The core jasmonate biosynthetic pathway is initiated when linolenic acid from plastidial membranes is converted by the successive action of lipoxygenase, allene oxide synthase and allene oxide cyclase into the precursor 12-oxo-phytodienoic acid (OPDA). This compound is then further reduced by OPDA-reductase 3 (OPR3) followed by three rounds of ß-oxidation to form the inactive pro-hormone jasmonic acid (JA) (Schaller and Stintzi 2009). Among many possible modification routes described for JA (Wasternack and Hause 2013), its conjugation to isoleucine by Jasmonate Resistant 1 (JAR1) constitutes the critical activation step, and resulting jasmonoyl-isoleucine (JA-Ile) acts as the master regulator of most JA responses in higher plants (Campos et al. 2014; Heitz et al. 2016). This occurs when JA-Ile promotes assembly of the F-box protein CORONATINE INSENSITIVE 1 (COI1) with one of numerous JASMONATE-ZIM-domain (JAZ) repressor proteins and disrupts their transcription-repressing function at promoter regions of target genes. Upon ligand-dependent co-receptor assembly, JAZ are tagged by ubiquitin E3 ligase complexes before proteolytic degradation, allowing transcription of JA-responsive genes to proceed and execute the numerous adaptation responses (Chini et al. 2007; De Geyter et al. 2012; Thines et al. 2007).

In this mechanism, the levels and dynamics of JA-Ile are of crucial importance to direct a timely orchestration of transcriptional cascades (Du et al. 2017; Hickman et al. 2017). Many biotic and abiotic stress conditions induce accumulation of JA/JA-Ile and presumed catabolic derivatives and only recently have new metabolic relationships been established within this hormone family (Heitz et al. 2016; Koo 2018). In particular, metabolic studies in Arabidopsis have revealed the existence of two enzymatic JA-Ile turnover routes that are induced along with biosynthetic and signaling pathways (Additional file 1: Figure S1). The first one consists in a two-step oxidation mediated by three cytochromes P450 of the 94 family (CYP94B1, B3 and C1) and the resulting 12OH-JA-Ile and 12COOH-JA-Ile products have strongly reduced capacity to promote COI1-JAZ co-receptor formation (Aubert et al. 2015; Heitz et al. 2012; Kitaoka et al. 2011; Koo et al. 2011, 2014). They are therefore considered as largely inactive derivatives. The second JA-Ile elimination pathway consists in conjugate cleavage by IAR3 and ILL6, two members of a multi-functional amido-hydrolase family, that release JA from JA-Ile, but also 12OH-JA from 12OH-JA-Ile (Widemann et al. 2013; Woldemariam et al. 2012; Zhang et al. 2016). Loss and mostly gain-of-function experiments have established that these enzymes deplete specifically JA-Ile hormone pools to attenuate JA signaling and contribute to the proper regulation and termination of JA-mediated processes. Despite of their importance, the nature and regulation of JA-Ile turnover pathways have not been investigated in crop species and may have significant impact on some agricultural traits with regards to stress tolerance. Rice is particularly suited for such a purpose as a model cereal and a major food crop. Recent research has identified candidate genes for most components of JA biosynthetic and signaling pathway and several features have been addressed functionally (Dhakarey et al. 2016; Liu et al. 2015; Riemann et al. 2015). Functions were described for example in photomorphogenesis (Svyatyna et al. 2014), spikelet development (Cai et al. 2014; Liu et al. 2017), control in the transition between juvenile and adult phases (Hibara et al. 2016), defense against insects (Lu et al. 2015; Ye et al. 2012) and pathogens (Riemann et al. 2013; Yamada et al. 2012). In addition, significant impacts of the JA pathway on abiotic stress tolerance were reported in this species (Dhakarey et al. 2016). The most documented case is probably salt stress resilience for which JA signaling seems to be detrimental, based on several reports. We showed previously that a JA-deficient rice mutant performs better upon salt exposure than wild-type (Hazman et al. 2015). Similarly, suppressing (OsJAZ9) or enhancing (OsJAZ8) salt-induction of JAZ repressors improves tolerance to this stress (Peethambaran et al. 2018; Wu et al. 2015). This data supports a previous report by Kurotani and co-workers (Kurotani et al. 2015a) showing that a rice line overexpressing CYP94C2b, a putative ortholog of the JA-Ile-oxidase CYP94C1 in Arabidopsis displays enhanced survival upon salt exposure. This notion was further extended by the finding of a positive correlation between expression levels of this gene and salt tolerance in an extensive collection of tolerant rice lines (Kurotani et al. 2015b), suggesting a possible link between JA-Ile catabolism and salt stress tolerance.

In the present work, we explored rice JA-Ile catabolism under two distinct stresses known to trigger JA biosynthesis and signaling, namely mechanical wounding used as a proxy for attack by chewing insects, and salt exposure as a relevant abiotic stress that increasingly threatens rice productivity under agricultural conditions (Shrivastava and Kumar 2015). We established detailed kinetic jasmonate profiles in seedlings, including catabolic derivatives, and examined the requirement of the conjugating enzyme JAR1 for each stress. We next mined the rice genome to explore the two known gene families, *CYP94* and amido-hydrolase (*AH*), in which some Arabidopsis members encode JA-Ile-metabolizing activities as described above. We identified through phylogenic analysis and gene expression studies candidate rice members that could potentially encode such activities. By recombinant protein expression, we were able to demonstrate for one CYP94 and one AH JA-Ile oxidation or cleaving activities, respectively. The study sheds light onto new players of JA hormone turnover and increases our understanding of JA-Ile homeostasis in rice.

## Results

### Occurrence and Diversity of *CYP94* and Amidohydrolase (*AH*) Genes in the Rice Genome

**Fig. 1:**
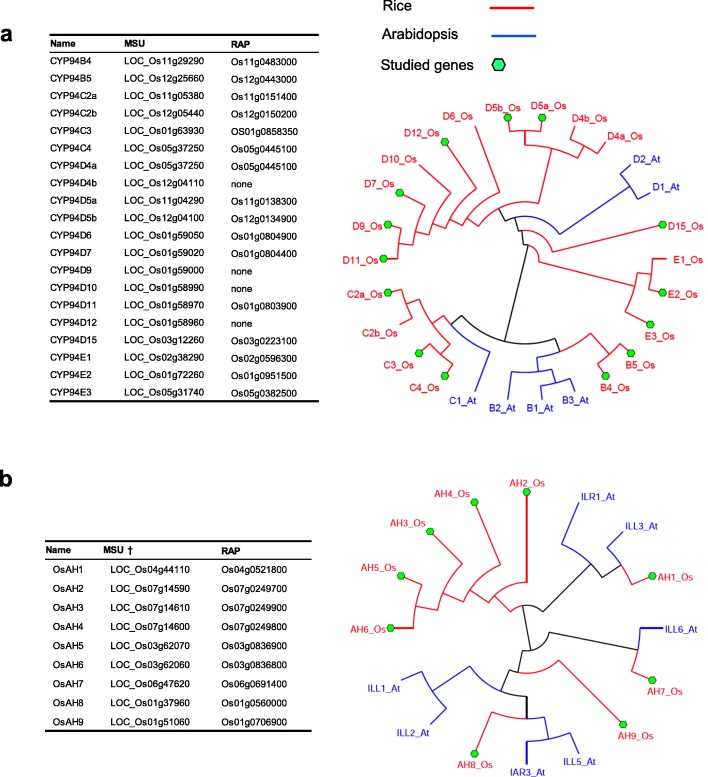
Occurrence and phylogeny of OsCYP94 and OsAH predicted proteins. Predicted proteins sequences of OsCYP94 (**a**, 20 proteins) and OsAH (**b**, 9 proteins) were retrieved from MSU rice genome project (http://rice.plantbiology.msu.edu/) and (RAPdb (http://rapdb.dna.affrc.go.jp). Respective loci numbers are indicated. Sequences were analyzed along with Arabidopsis sequences (shown in blue) using the Phylogeny suite (Dereeper et al. 2008) and DENDROSCOPE v3 (Huson and Scornavacca 2012)

The rice complement of JA-Ile catabolic genes/enzymes was explored, based on similarity search using known Arabidopsis sequences. *CYP94* gene family was reported previously to contain about 18 distinct genes in the sequenced genome of Nipponbare rice (Widemann et al. 2015). Here we report 20 *OsCYP94*-related loci from the MSU database, for which not all have counterparts in the RAP database. OsCYP94 predicted proteins display a particular subclade enrichment. Like Arabidopsis, rice bears no subclade A member (Fig. 1a) and exhibits only 2 subclade B proteins, OsCYP94B4 and OsCYP94B5 along with 4 potential members in subclade C, OsCYP94C2a, OsCYP94C2b*, Os*CYP94C3 and OsCYP94C4. In these latter two subclades, JA-Ile oxidases have been characterized in Arabidopsis (Heitz et al. 2012; Kitaoka et al. 2011; Koo et al. 2011). Subclade D and a new grass-specific subclade E, both of unknown function, are particularly diversified in rice with 11 and 3 members, respectively. These data indicate considerable CYP94 expansion and diversification in the rice genome.

Rice amidohydrolase (AH) gene family was similarly investigated and 9 predicted proteins were identified. Figure 1b shows their phylogenetic relationships with the 7 Arabidopsis AH protein sequences (Rampey et al. 2004). Five members (OsAH2 through OsAH6) cluster with AtILR1 that prefers auxin-amino acid conjugates as substrates (Zhang et al. 2016). Two other isoforms, OsAH1 and OsAH9 were more distantly related to previously characterized enzymes. The closest rice homolog of AtIAR3, the conserved bifunctional AH cleaving both JA-amino acid and indole acetic acid-amino acid conjugates (Widemann et al. 2013; Zhang et al. 2016) was OsAH8 (78/64% similar/identical). Finally, OsAH7 protein sequence was closely related (75/63% similar/identical) to JA-Ile-specific AtILL6.

### Specific *CYP94* and *AH* Gene Isoforms Display Dynamic Expression upon Wound Stress

To investigate stress responsiveness of members in both gene families, expression studies were conducted in leaves using kinetic responses to wounding. Specific primer pairs were designed for genes with unique 3’UTR sequences available from databases, comprising 14 *OsCYP94* genes and all 9 *OsAH* genes (Fig. 1). This criterion was met for *CYP94C2a*, but not for the very similar (91%) and formerly reported *CYP94C2b* gene (Kurotani et al. 2015a) for which no specific primer pair could be designed. Most analyzed *OsCYP94* genes exhibited wound-induced expression, either as a rapid and transient pulse peaking at 0.5 h post-wounding (hpw), as for *CYP94B4*, *CYP94B5*, *CYP94C2a*, *CYP94C3*, *CYP94C4*, or as a steady increase over the 6 h period studied, for *CYP94D5a*, *CYP94D7*, *CYP94D9*, *CYP94E2* (Additional file 2: Figure S2). However, overall expression levels were extremely variable between genes, and ranged over 3 orders of magnitude. Despite of detectable wound-triggered changes, some isoforms displayed very low expression levels, like *CYP4B4*, *CYP94C3*, *CYP94D5*, *CYP94D11*, *CYP94D12*, *CYP94D15*, and *CYP94E2*, and were not further studied.

A similar approach was taken to visualize *AH* gene expression. Slow increase upon wounding was recorded for *AH1*, *AH2*, *AH3*, *AH6*, and *AH8* transcripts, with maximal expression at later time points (Additional file 3: Figure S3). Because of rapid accumulation of JAs within the first hour after leaf wounding in Arabidopsis (Glauser et al. 2008; Heitz et al. 2012; Widemann et al. 2013), we speculated that genes whose expression was rapidly induced to high levels by wounding would be best candidates to participate in rice JA metabolism. AH7 combined a rapid and transient induction peaking at 0.5 hpw and relatively high expression, whereas AH8 displayed the highest basal expression and moderate induction by 3 hpw.

**Fig. 2:**
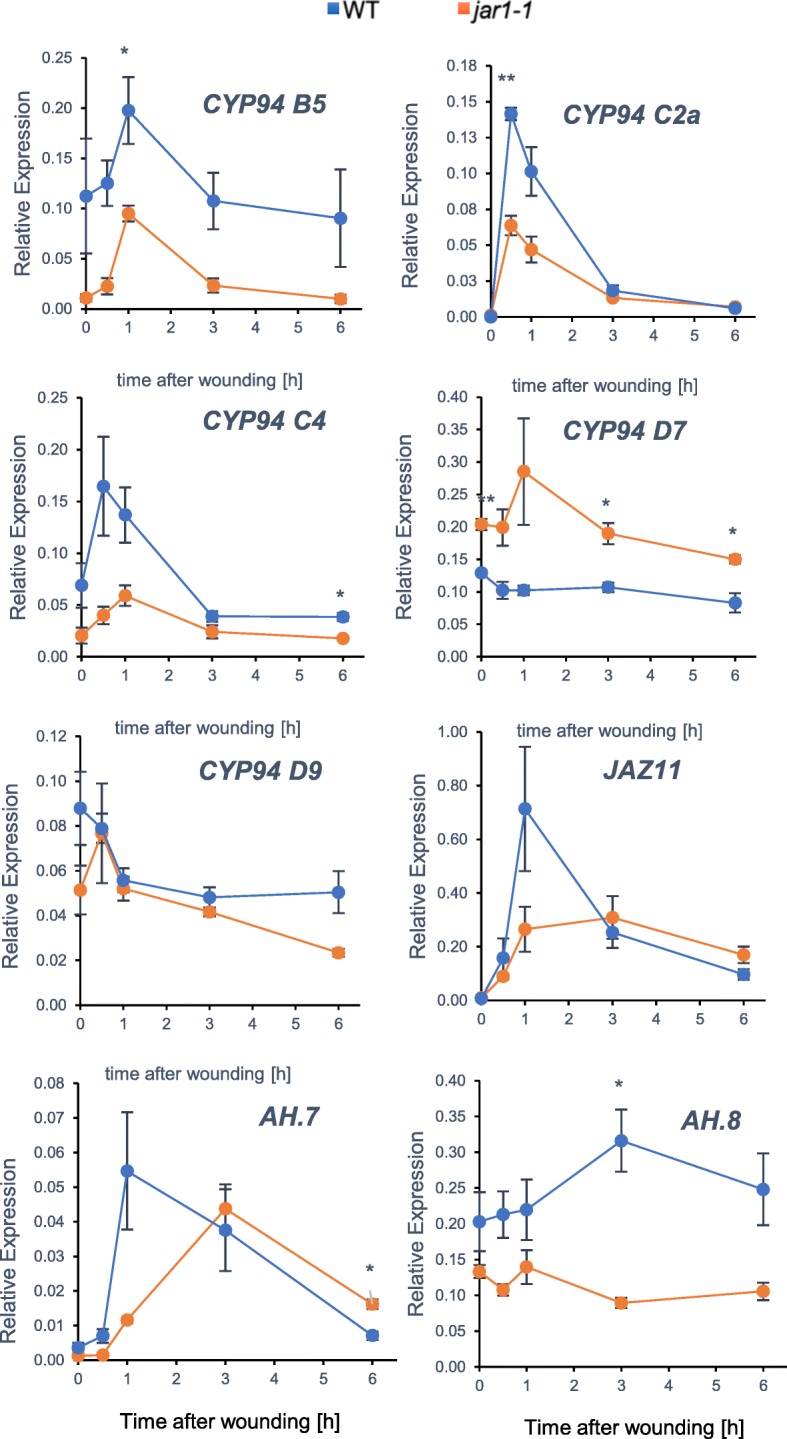
Selected *OsCYP94* and *OsAH* gene expression upon leaf wounding in WT and *jar1–1* seedlings. Ten-day old (seedlings were submitted to leaf wounding and 2nd leaf was harvested at the indicated time points. Total RNA was submitted to RT-qPCR to reveal expression of indicated genes. Relative expression was determined using *OsGAPDH* as reference gene. Values are means and SD from 3 independent biological replicates

We next addressed the transcriptional response of members in *OsCYP94* or *OsAH* gene families, with focus on subclades predicted to encode JA-Ile metabolizing enzymes, in a parallel analysis of WT and *jar1–1* mutant plants impaired in the major conjugating enzyme forming JA-Ile (Svyatyna et al. 2014). Most genes analyzed were induced by wounding to a lower extent but with similar kinetics in *jar1–1* and in WT plants, as exemplified by *JAZ11* gene used as a JA-Ile-regulated control (Fig. 2). This was particularly the case for *OsCYP94B5*, *C2a*, *C4*, and to a lesser extent for *D7* and *D9*, indicating that their full induction depends on JA-Ile synthesis and signaling. In contrast, in *jar1–1 OsAH7* was quantitatively similar to WT but peaked later (3 hpw), and *OsAH8* expression was only marginally affected by *jar1–1* mutation. These data identify *CYP94B5*, *C2*, *C4* on the one hand, and *AH7* and *AH8* on the other hand as highly expressed and/or and wound-responsive encoding potential JA-Ile catabolizing enzymes in rice.

### Wounding Induces Strong, Partially JAR1-Dependent JA Accumulation and Catabolism in Rice

**Fig. 3:**
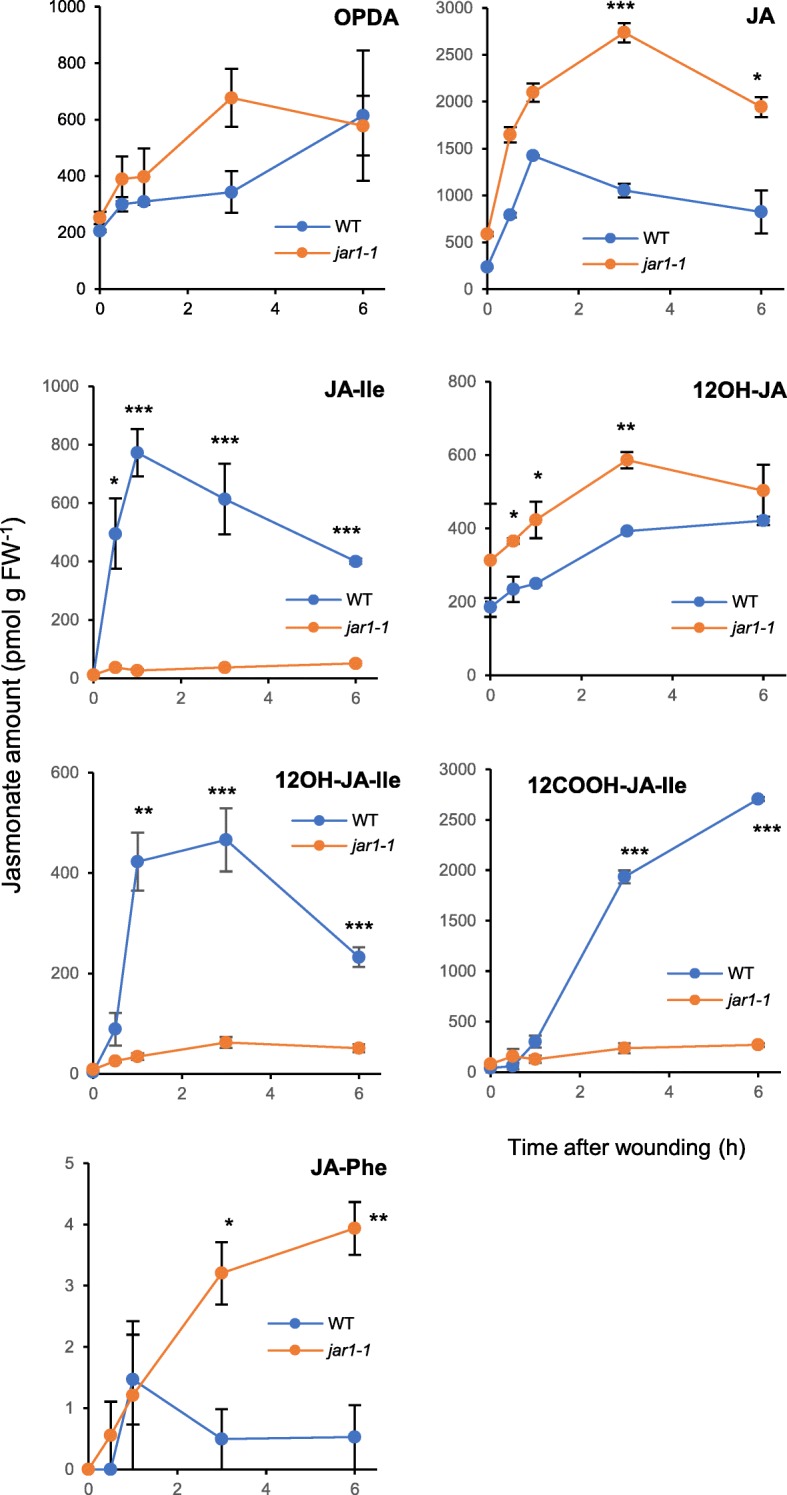
Kinetic analysis of jasmonate accumulation in WT and *jar1–1* plants upon mechanical leaf wounding. Ten-days old Nihonmasari WT and *jar1–1* seedlings (same batch as in Fig. 2) were submitted to leaf wounding and 2nd leaf was harvested at the indicated time points. Leaves were extracted for jasmonate determination by UPLC-MS/MS. OPDA: 12-oxo-phytodienoic acid; JA: jasmonic acid; JA-Ile: jasmonoyl-isoleucine; 12OH-JA: 12-hydroxy-jasmonic acid; 12OH-JA-Ile: 12-hydroxy-jasmonoyl-isoleucine; 12COOH-JA-Ile: 12-carboxy-jasmonoyl-isoleucine; JA-Phe: jasmonoyl-phenylalanine. Values are mean and SD of 3 independent biological replicates

Wounded plant material was then subjected to targeted JA profiling by UPLC-MS/MS. We quantified evolution of JAs upstream and downstream of bioactive JA-Ile, including OPDA, JA, JA-Ile and the catabolites 12OH-JA-Ile, 12COOH-JA-Ile and 12OH-JA, these latter deriving from JA-Ile-oxidizing and -cleaving activities. Resulting profiles are shown in Fig. 3. OPDA and JA accumulated upon wounding as expected, and were more abundant in *jar1–1* than in WT, consistent with a block in JA-consuming JAR1 activity. JA-Ile abundance displayed a typical sharp increase peaking at 1 hpw that readily declined afterwards (Heitz et al. 2012; Wakuta et al. 2011), and its wound-induced accumulation was efficiently abolished in *jar1–1*. Its oxidized derivative 12OH-JA-Ile paralleled JA-Ile profiles whereas second catabolite 12COOH-JA-Ile accumulated with more delayed kinetics. These results are in accordance with the concomitant induction of *OsCYP94B5*, *C2a* or *C4* expression (Fig. 2) as putative JA-Ile oxidases. Furthermore, in response to wounding the abundance of oxidized conjugates was largely dependent on JAR1. Interestingly, 12OH-JA, which is known to be mostly generated by the sequential action of the CYP94-AH pathways in wounded Arabidopsis leaves (Smirnova et al. 2017; Widemann et al. 2013) (Additional file 1: Figure S1) is more abundant in *jar1–1*, suggesting a minor contribution of conjugated intermediates for its synthesis in wounded rice. Finally, JA-Phe, a minor conjugate reported in stressed Arabidopsis leaves (Kitaoka et al. 2014; Widemann et al. 2015) accumulated in trace amounts, that were more abundant in *jar1–1* than in WT in wounded rice leaves. In contrast, no signal was detected for the following JA-amino-acid conjugates: JA-Cys, JA-Gly, JA-Glu, JA-Pro, JA-Trp or JA-Tyr.

### Responses of JA-Ile Catabolic Genes to Salt Stress

**Fig. 4:**
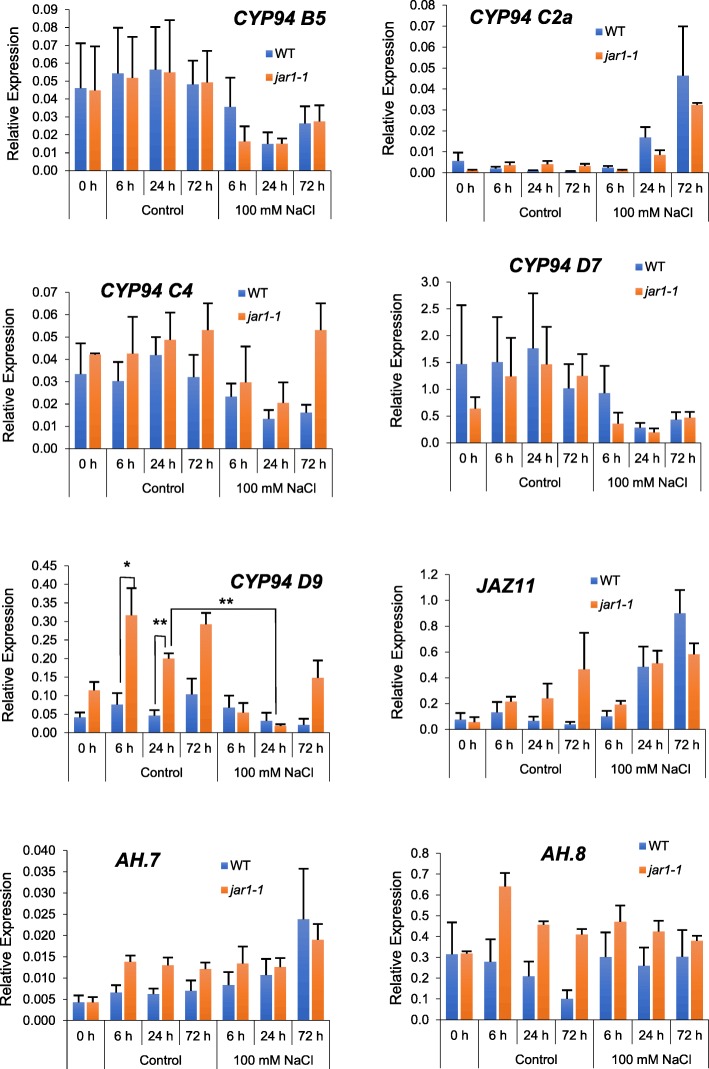
Selected *OsCYP94* and *OsAH* gene expression upon salt stress in WT and *jar1–1* seedlings. Ten-days old seedlings were submitted to 100 mM NaCl stress and 2nd leaf was harvested at the indicated time points. Total RNA was submitted to RT-qPCR to reveal expression of indicated genes. Relative expression was determined using *OsUbiquitin10 (OsUBQ10)* and *OsZCF61* as reference genes. Values are means and SD from 3 independent biological replicates

Because some impacts of the JA pathway on salt stress response have been reported (Hazman et al. 2015; Peethambaran et al. 2018), we examined the transcriptional behavior of the same set of selected *CYP94* and *AH* genes after seedling exposure to 100 mM NaCl stress. The material eventually developed symptoms of wilting as described in Hazman et al. (2015). Leaves were harvested 6, 24 or 72 h after onset of stress and extracted RNA was submitted to RT-qPCR analysis. *CYP94B5* expression decreased to about half control values upon salt exposure, independently of JAR1 (Fig. 4). *CYP94C4* followed a similar trend in WT, but its expression was less affected in *jar1–1*. In contrast, *CYP94C2a* expression was strongly induced upon salt stress and this response was essentially maintained in *jar1–1*, similar to *JAZ11* used as a JA-Ile-regulated marker. *CYP94D7* was down-regulated by salt in a JAR1-independent manner, whereas *CYP94D9* displayed a higher expression in *jar1–1* control samples, that declined to WT levels in response to salt. Finally, *AH8* expression was unreactive to salt stress, with slightly higher expression in *jar1–1*. AH7 was found moderately upregulated by salt in WT, while its transcripts were slightly elevated in JAR1-deficient leaves. These data collectively show that salt stress induces specific changes in expression of *CYP94* and *AH* members and indicate a limited influence of JAR1-mediated signaling on these changes.

### Salt Stress Triggers JA Accumulation and Catabolism

**Fig. 5:**
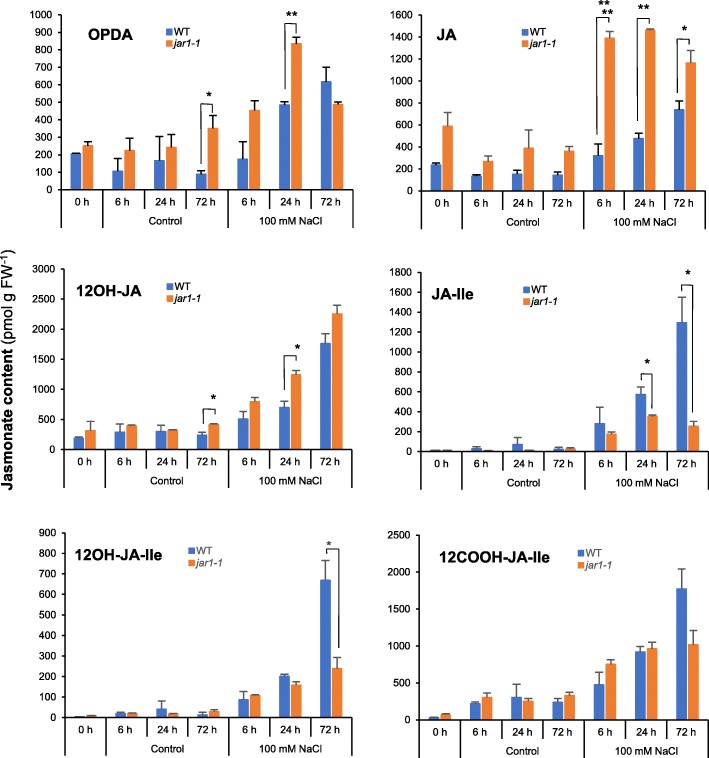
Kinetic analysis of jasmonate accumulation in WT and *jar1–1* plants upon salt stress in WT and *jar1–1* seedlings. Thirteen-days old Nihonmasari (WT) and *jar1–1* seedlings (same batch as in Fig. 4) were submitted to 100 mM NaCl stress and 2nd leaf was harvested at the indicated time points. Leaves were extracted for jasmonate determination by UPLC-MS/MS. OPDA: 12-oxo-phytodienoic acid; JA: jasmonic acid; JA-Ile: jasmonoyl-isoleucine; 12OH-JA: 12-hydroxy-jasmonic acid; 12OH-JA-Ile: 12-hydroxy-jasmonic acid; 12COOH-JA-Ile: 12-carboxy-jasmonoyl-isoleucine. Values are mean and SD of 3 independent biological replicates

Leaves from salt-exposed and control seedlings were profiled for JAs content. All compounds analyzed increased steadily in response to stress in WT plants, with maximal levels at the latest time point recorded, after 72 h of exposure (Fig. 5). Like for wounding, OPDA and JA levels were enhanced by salt at most time points, but to higher levels in *jar1–1* mutant compared to WT (Fig. 5). JA-Ile was very low in control plants but increased steadily in stressed WT material to about 1.4 nmol g FW^− 1^. Interestingly, lower but significant accumulation was also recorded in *jar1–1*. Accordingly, the JA-Ile catabolites 12OH-JA-Ile and 12COOH-JA-Ile increased in response to salt exposure, with a JAR1-dependence which decreased along the pathway from JA-Ile to 12OH-JA-Ile and 12COOH-JA-Ile (Fig. 5). Because in *jar1–1* JA-Ile still accumulated moderately, we examined transcript levels of the closely related homolog JAR2 that also conjugates JA to amino acids, including Ile (Svyatyna et al. 2014; Wakuta et al. 2011). As shown in Additional file 4: Figure S4A, *JAR2* expression was found to be salt-repressed both in WT and in *jar1–1* genotypes, making it unlikely that JAR2 contributes to salt-induced JA-Ile accumulation.

### Functional Analysis of CYP94 and AH Proteins

**Fig. 6:**
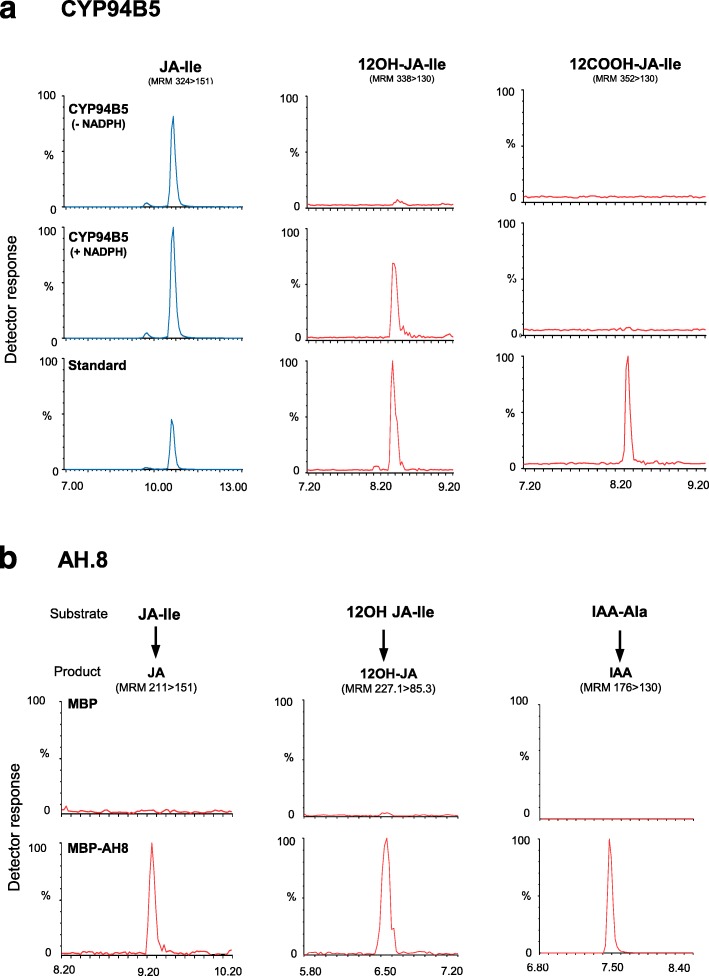
In vitro enzymatic activity of recombinant OsCYP94B5 and OsAH8. **a** Microsomes of OsCYP94B5-expressing yeast were prepared and incubated for 30 min with 100 μM JA-Ile (blue traces) in absence or in presence of NADPH cofactor. Expected oxidation products (red traces) were identified based on the retention time and detection in multiple reaction monitoring mode of authentic 12OH-JA-Ile and 12COOH-JA-Ile standards. **b** Recombinant his-tagged OsAH8 fused to Maltose Binding Protein (MBP) was affinity purified and incubated with 30 μM of either of the following amino-acid conjugates: JA-Ile, 12OH-JA-Ile or IAA-Ala. Respective cleavage products were searched for by detection based on retention time and multiple reaction monitoring mode of authentic JA, 12OH-JA or IAA standards. Analysis were performed in triplicates and one representative trace is shown

In an attempt to determine JA-Ile-metabolizing capacities of encoded proteins, selected genes were expressed in heterologous hosts. To this end, *CYP94B5*, *CYP94C2a* and *CYP94C4* cDNAs were cloned in pYeDP60 vector for expression in yeast. Microsome fractions were prepared as enzyme sources from yeast cells and incubated with synthetic JA-Ile before analyzing the formation of oxidation products by UPLC-MS/MS. As shown in Fig. 6a, microsomes from CYP94B5-expressing yeast yielded a clear signal matching characteristics of 12OH-JA-Ile standard compound, when incubation was performed in presence of NADPH cofactor. 12COOH-JA-Ile, the second oxidation product of AtCYP94C1 (Heitz et al. 2012) could not be detected in incubations. Experiments with microsomes of *CYP94C2*- or *CYP94C4*-transformed yeasts were unsuccessful in evidencing in vitro enzyme activity.

AH7 and AH8 coding sequences were cloned in expression vector pHMGWA, and expression of double-tagged 6xHis-Maltose Binding Protein (MBP)-AH was induced in bacterial cells. Fusion proteins of the expected sizes (His-MBP-AH8: 86.4 kDa; His-MBP-AH7: 92.7 kDa) could be isolated. His-MBP-AH8 was produced abundantly in soluble form and was purified by dextrin affinity chromatography followed by gel-filtration (Additional file 5: Figure S5a). His-MBP-AH7 expressed at lower abundance and was captured in bacterial lysates by immobilized metal affinity chromatography (IMAC) (Additional file 5: Figure S5b). Both proteins were next incubated with hormone-amino acid conjugates, typically JA- or auxin-amino acid conjugates, that were previously described as in vivo substrates of this class of enzymes (Rampey et al. 2004; Widemann et al. 2013; Zhang et al. 2016). Recombinant OsAH7 and OsAH8 were incubated with either JA-Ile, or with its oxidized derivative 12OH-JA-Ile, or with the auxin conjugate IAA-Ala. Corresponding unconjugated hormonal compounds were searched for as evidence of cleaving activity. No activity could be recorded for OsAH7 with either substrate (not shown). In contrast, chromatograms shown in Fig. 6b illustrate that OsAH8, similarly to its Arabidopsis homolog AtIAR3, is able to generate free JA, 12OH-JA and IAA from their respective conjugated substrates.

## Discussion

Jasmonate signaling is governing major aspects of plant development and adaptation to environmental stress. Recent reports have revealed positive and negative contributions of JA signaling to biotic and abiotic threats (Hazman et al. 2015; Peethambaran et al. 2018; Riemann et al. 2013, 2015; Wu et al. 2015), calling for a better understanding of hormonal regulation. Catabolic processes affecting bioactive JA-Ile hormone homeostasis have been elucidated in Arabidopsis and shown to attenuate JA signaling (Heitz et al. 2012; Koo et al. 2011; Koo and Howe 2012; Widemann et al. 2013; Zhang et al. 2016). The precise actors and mechanisms of JA-Ile turnover were unknown in rice, but conservation of the oxidative pathway in this species was suggested by the correlation of OsCYP94C2b overexpression with higher *in planta* JA-Ile oxidation, leading to increased salt tolerance (Kurotani et al. 2015a, 2015b). Here we explored the rice *CYP94* and *AH* gene families, in a search for orthologs of the Arabidopsis JA-Ile oxidation and deconjugation enzymes (Heitz et al. 2016; Koo and Howe 2012). Similarity search readily identified large gene families; strikingly, subclades *B* and *C* of *CYP94* encoding suspected JA-Ile oxidases only represented a minor portion of the *OsCYP94* gene family, in contrast to subclade *D* of unknown activity that displays up to 10 members. The additional occurrence of 3 subclade *E* genes illustrates the high diversification of CYP94 genes/proteins in rice and questions the existence of distinct enzymatic activities. In contrast, the *AH* gene family, with 9 members in rice had a comparable complexity to the 7-member Arabidopsis family.

To identify stress-responsive members, we examined transcriptional behavior of a subset of *OsCYP94* and all *OsAH* genes, in response to leaf wounding or salt exposure, two stresses known to cause rapid/massive, or slower changes in JA metabolism, respectively. Only members of the *CYP94B* and *C* subclades were found to display a stereotypical expression peak at 0.5–1 hpw that parallels the transient accumulation of JA-Ile, their presumed substrate, upon wounding (Heitz et al. 2012; Wakuta et al. 2011). Despite of sharing this profile, *CYP94B4* and *CYP94C3* have very low expression and likely are not major players of JA-Ile oxidation in wounded tissues. In contrast, *OsCYP94B5*, *C2a* and *C4* displayed much higher expression, and similarly to Arabidopsis characterized *CYP94B1*, *B3* and *C1* genes (Heitz et al. 2012), their induction was partially dependent on JA-Ile biosynthesis, as evidenced in the *jar1–1* mutant. Quantitative, kinetic JA profiling was performed to reveal the characteristics of JA/JA-Ile catabolism in rice. Main features reported in Arabidopsis were conserved, with slower accumulation of its two oxidized derivatives. Therefore, OsCYP94B5, C2a and C4 are the prime candidates for wound-induced JA-Ile turn-over in rice. All 3 Ile-conjugates were nearly absent in *jar1–1*, but the minor conjugate JA-Phe was more abundant in this mutant, indicating it is produced by another conjugating enzyme, possibly boosted by the enhanced accumulation of JA precursor. JAR2, the closest JAR homolog in the GH3 family of rice, is unable to form JA-Phe in vitro (Svyatyna et al. 2014), and we established that its transcript levels decreased upon wounding in our experiments (Additional file 4: Figure S4B). Therefore, an unknown enzyme, possibly in the GH3 family may be involved in JA-Phe formation (Wakuta et al. 2011). In the *AH* family, *OsAH7* and *OsAH8* behaved also similarly to their respective Arabidopsis *AtILL6* and *AtIAR3* closest homologs (Widemann et al. 2013), with a stable but high expression for *OsAH8*, and a dynamic pulse with weaker expression for *OsAH7*. These features do not allow to draw firm conclusions as to which AH contributes most to JA-Ile cleavage.

Transcriptional responses to salt exposure were contrasted. Only *OsCYP94C2a* was induced, similarly to *OsJAZ11*, and accompanied the increase in JA-Ile and its catabolites, whereas *OsCYP94B5* and *OsCYP94C4* were essentially down-regulated, as well as *OsCYP94D7* and *D9*. Therefore, *OsCYP94C2a* is the most likely actor of JA-Ile oxidation upon salt-stress, lending support to earlier data by Kurotani et al. (2015a, 2015b), who identified in a genetic screen the related *OsCYP94C2b* as a major single-gene contributor to salt tolerance. *OsCYP94D7* and *D9* decrease could underlie the need to shutdown a distinct enzyme activity upon salt response. *OsAH7* and *OsAH8* transcripts only fluctuated marginally. As upon wounding, JA was hyperaccumulated in *jar1–1* in salt-reacting leaves, indicating reduced JA metabolization, but Ile-conjugates were less reduced in this latter material, suggesting the contribution of another conjugating enzyme than JAR1. JAR2, the closest candidate to fulfill this function, is able to form JA-Ile in vitro (Svyatyna et al. 2014), but its contribution to salt-induced JA-Ile accumulation is not supported by the down-regulation of its transcripts. Currently, we are lacking information on alternative enzyme activities in the GH3 families, hence, more efforts are needed to clarify JAR1-independent JA conjugation. The fact that oxidized derivatives are less affected by *jar1–1* mutation than JA-Ile may indicate that they undergo less deconjugation in this background and are therefore more stable.

Lastly, we set out to characterize directly enzyme activities of recombinant proteins. Absence of activity in OsCYP94C2a and C4 in yeast microsomes may be attributed to inactive conformation and/or insufficient expression. When carbon monoxide spectra of microsomes obtained from yeast transformed with OsCYP94C2a and C4 were recorded, a peak at about 420 nm rather than 450 nm was observed, suggesting the presence of misfolded heme-containing proteins (Luthra et al. 2011).

Our failure to assay recombinant OsCYP94C2a enzyme activity, combined with the absence of information on stress-induced expression of the closely related *OsCYP94C2b* gene (Kurotani et al. 2015a) limits the knowledge of the relative contribution of these two isoforms to JA-Ile turnover. Affinity-purified OsAH7 also proved inactive, possibly due to mis-folding or hindrance by the MBP fusion. This is reminiscent of AtILL6, its Arabidopsis counterpart that is also notoriously difficult to express/assay (Widemann et al. 2013). However, for two enzymes, we could demonstrate JA-Ile-metabolizing activity. OsCYP94B5 displays JA-Ile oxidase activity in vitro, consistent with its Arabidopsis CYP94B orthologs (Heitz et al. 2012; Kitaoka et al. 2011; Koo et al. 2014). Rendering *CYP94B5* expression salt-responsive for enhanced JA-Ile oxidative inactivation could be an attractive strategy to engineer salt-tolerance in rice. OsAH8 could be obtained in the active form, and its ability to cleave JA-aa and IAA-aa conjugates was shared with the bi-functional Arabidopsis IAR3 (Widemann et al. 2013; Zhang et al. 2016).

## Conclusions

Our work sheds light on rice *CYP94* and *AH* gene families, in a context of hormone conjugate metabolism. CYP94 is so far limited to jasmonate substrates while some AH also readily catalyze cleavage of auxin conjugates (Zhang et al. 2016). The present evidence suggests that the JA-Ile catabolic network architecture is conserved in rice, both in terms of phylogeny and catalytic capacities: the closest homologous proteins display similar activities to enzymes characterized in Arabidopsis, but some unexpected regulations were also recorded. These findings and tools pave the way for stress-specific manipulation of JA-Ile catabolism for optimized output of the JA signaling pathway under various stresses.

## Materials and Methods

### Phylogenetic Analysis

Dr. D. Nelson (University of Tennesse, USA) kindly provided the names of CYP94 isoforms. The full length amino acid of rice cytochrome P450 (CYP94) and amidohydrolase (AH) sequences were extracted from rice databases MSU (http://rice.plantbiology.msu.edu) and RAP (http://rapdb.dna.affrc.go.jp) via blasting CYP94 and AH Arabidopsis protein sequences. CYP94 sequences were matched with sequences provided by D. Nelson. All sequences were then manually curated and loci identified in rice genome databases. A total of deduced 26 CYP94 and 16 AH full-length amino acid sequences were analyzed using the phylogeny suite (Dereeper et al. 2008) and DENDROSCOPE v3 (Huson and Scornavacca 2012).

### Plant Materials, Growth and Stress Conditions

In this study, *Oryza sativa* L. ssp. japonica cv. Nipponbare was used as the wild type, and *jar1–1* (Riemann et al. 2008) was used as mutant. The caryopses were dehusked and surface sterilized according to (Hazman et al. 2015) and sowed on 0.4% phytoagar containing 5% MS salts (Duchefa, Haarlem, The Netherlands) and incubated for 7 or 10 days in a culture room under continuous light at 25 °C. For wounding stress, 10-day old seedlings were subjected to manual wounding using either metal forceps (Additional file 2: Figure S2, Additional file 3: Figure S3) or scissors (Fig. 2 and Fig. 3) to produce consistent wounds along the second leaf (5 and 7 wounds/ leaf, respectively). Wounded leaves were harvested kinetically (0, 0.5, 1, 3 and 6 h after wounding) in 3 replicates and then stored at − 80 °C for quantitative gene expression analysis and jasmonate profiling. For salt stress, plant cultivation and treatments were accomplished according to Hazman et al. (2015). Briefly, surface sterilized caryopses of both WT and *jar1–1* mutants were pre-germinated on 0.4% phytoagar for 7 days under continuous light of 120 μmol m^− 2^ s^− 1^ at 25 °C. The seedlings were transferred to microtubes with cut ends that were inserted on floating racks placed to a cylindric glass container containing 5% MS medium as nutrient solution for extra 3 days. Subsequently, the solution was replaced by the same solution (control time course) or the same solution containing NaCl (100 mM). Leaves of both control and stressed plants were harvested kinetically (0, 6, 24 and 72 h after transferring into final solution) in multiple replicates and then stored at − 80 °C for profiling of gene expression or jasmonate content.

### Total RNA Extraction and Quantitative Real-Time PCR

For data shown in Additional file 2: Figure S2, Additional file 3: Figure S3, total RNA was isolated from the shoots of control, wounded and salt treated plants using Trizol Reagent (Molecular Research Center, Cincinatti, USA) according to the manufacturer’s instructions. The cDNA synthesis was performed with cDNA synthesis kit (Invitrogen, Carlsbad, USA) using 1 μg total RNA as a template. RT-qPCR (Reverse Transcriptase – quantitative PCR) was performed on 10 ng cDNA with a SYBR green dye protocol using LightCycler 480 II instrument (Roche Applied Science, Penzberg, Germany) as follows: 95 °C for 3 min, and 40 cycles (95 °C for 15 s, annealing at 66 °C for 30 s and extension at 72 °C for 30 s). The gene expression levels in three biological replicates were calculated using the ∆∆^Ct^ method.

For data shown in Fig. 2 and Fig. 4, RNA-extraction was performed using the innuPREP Plant RNA KIT (Analytik Jena AG, Jena, Germany) according to instructions provided by the manufacturer. The RNA was transcribed via a first strand cDNA using MuLV reverse transcriptase (New England Biolabs, Frankfurt, Germany) and oligo dT-primers in a two-step reaction. Quantitative analysis was performed on a CFX Touch real-time PCR system (Bio-Rad, Munich, Germany) according to the protocol of Svyatyna et al. (2014). The primer sequences for the genes of interest and reference genes are listed in Additional file 6: Table S1.

### Recombinant Heterologous Expression of OsCYP94B5 in Yeast and OsAH8 in Bacteria

Coding sequence of *OsCYP94B5*, *OsCYP94B4* and *OsCYP94C2a* was generated by PCR amplification from rice genomic DNA cultivar Nipponbare. The forward and reverse primer sequences are given in Additional file 6: Table S1. The amplified PCR product was initially cloned in pGEM-T easy vector according to manufacturer instructions (Promega) and then sequenced to verify error-free insert that was cloned in the *Bam*H1 and *Eco*R1 sites of the yeast expression vector pYeDP60. OsCYP94B5, OsCYP94B4 and OsCYP94C2a proteins were produced in yeast optimized heterologous system as described in Heitz et al. (2012). P450 expression and quality control was performed by differential spectrophotometry as described in Gavira et al. (2013).

For heterologous expression of OsAH7 and OsAH8, open reading frame sequence deleted of the 69 (AH7) or 78 (AH8) N-terminal signal peptide-encoding nucleotides was amplified using a mixture (8:1) of hotstart Taq Polymerase and Phusion Taq Polymerase (Thermo Scientific, Illkirch-Graffenstaden, France) prior to the cloning in the pDONOR-Zeo vector (Thermo Scientific) in the DH5α *Escherichia coli* strain. Inserts with error-free sequences were recombined into the expression vector pHMGWA. Plasmid was further transformed into the *E. coli* Rosetta 2 (DE3) strain (Merck, Darmstadt, Germany). Ice-cold bacterial pellets from isopropyl ß-D-1-thiogalactopyranoside-induced (0.5 mM) cultures were collected and kept frozen until use. For AH7 purification, pellet was thawed on ice for 30 min while resuspending in lysis buffer (50 mM Tris–HCl pH 7.5, 300 mM NaCl, 20 mM imidazole, 3 mg/ml lysozyme) to an OD_600_ of 20 units. Bacteria were lysed by pulsed-sonication on ice for 4 min. Lysate was clarified by centrifugation, supernatant was filtered and diluted with 1 vol buffer (Tris-HCl 50 mM pH 8, NaCl 300 mM, glycerol 5%) before loading on a His-Trap FF (1 ml capacity) column mounted on an Äkta purifier system (GE Healthcare, Velizy, France) and equilibrated with the same buffer. His-tagged protein was eluted with equilibration buffer containing 500 mM imidazole. For AH8 purification, bacterial pellet was resuspended in 1 x Phosphate Buffered Saline (PBS) pH 7.3 buffer to an OD_600_ of 20 units before lysis by pulsed sonication on ice for 4 min. Clarified lysate was loaded on a MBP-Trap (1 ml capacity) column pre-equilibrated with PBS solution and protein was eluted with equilibration buffer complemented with 10 mM maltose. After analysis by SDS–PAGE, selected fractions were concentrated to 500 μL and loaded on a Superdex 200 10/300 column equilibrated in 1x PBS pH 7.3. Concentration of proteins of interest was estimated via quantification with Bradford reagent using a bovine serum albumin calibration series.

### Enzyme Assays and Analysis

For CYP94 assay, microsome incubations were performed as described in Widemann et al. (2015) with the following modifications: JA-Ile substrate concentration was 50 μM and the reaction was stopped with 150 μL methanol containing 0.2% acetic acid. The assays were centrifuged at 10000 g and the supernatant was used for LC-MS/MS analysis.

Amidohydrolase assay was performed in 200 μL as described in Widemann et al. (2013) using 10 μg affinity-purified protein and 30 μM substrates (JA-Ile, 12OH-JA-Ile or IAA). Both types of enzyme activities were analyzed by LC-MS as described in Widemann et al. (2015) on a Waters Quattro Premier XE (Waters, Mildorf, MA USA) instrument, using the following detection parameters: in negative mode: JA 209 > 59; 12OH-JA 225 > 59; 12OH-JA-Ile 338 > 130; 12COOH-JA-Ile 352 > 130; in positive mode: JA-Ile 324 > 151; IAA-Ala 247 > 130; IAA 176 > 130.

### Jasmonate Profiling

Jasmonate profiling of rice leaves was performed as follows: about 50–100 mg frozen plant material was extracted with 8 volumes of ice-cold extraction solution (MeOH:water:acetic acid 70:29:0.5) containing 9,10-dihydro-JA and 9,10-dihydro-JA-Ile as internal standards for workup recovery. Grinding was performed with a glass-bead Precellys tissue homogenizer (Bertin Instruments, Montigny-le-Bretonneux, France) in 2 mL screw-capped tubes. After 30 min incubation at 4 °C on a rotating wheel, homogenates were cleared before concentration under a stream of N_2_ and overnight conservation at − 20 °C. After a second centrifuge step, extracts were submitted to LC-MS/MS analysis on an EvoQ Elite LC-TQ (Bruker, Palaiseau, France). Column and chromatographic conditions were as described in Smirnova et al. (2017). Absolute quantifications were achieved by comparison of sample signals with dose–response curves established with pure compounds. Compound specific detections were performed in Multiple Reaction Monitoring Mode (MRM) using transitions described in Additional file 7: Table S2.

### Statistical Analysis

All statistical analysis were performed using InfoStat 2015d (http:// www.infostat.com.ar). Comparisons of sample means were per- formed by one-way analysis of variance (*P* < 0.05 or *P* < 0.01) and Tukey’s post-hoc multiple comparisons tests (*P* < 0.05 or *P* < 0.01), and significant differences of means were determined.

## Additional files

Additional file 1: **Figure S1.** Arabidopsis simplified jasmonate metabolic pathway. JA-Ile oxidative and cleavage pathways are shown in yellow and blue backgrounds, respectively. (DOCX 48 kb)

Additional file 2: **Figure S2.** *OsCYP94* gene expression upon leaf wounding. Ten-days old seedlings were submitted to leaf wounding and 2nd leaf was harvested at the indicated time points. Total RNA was submitted to RT-qPCR with specific primers for selected *OsCYP94* genes (as indicated in Fig. 1). Relative expression was determined using Actin as reference gene. Values are means and SD from 3 replicate determinations. (DOCX 72 kb)

Additional file 3: **Figure S3.** *OsAH* gene expression upon leaf wounding. Ten-days old seedlings were submitted to leaf wounding and 2nd leaf was harvested at the indicated time points. Total RNA was submitted to RT-qPCR with *AH*-specific primers. Relative expression was determined using Actin as reference gene. Values are means and SD from 3 replicate determinations. (DOCX 57 kb)

Additional file 4: **Figure S4.** Expression profile of *OsJAR2* upon salinity stress and leaf wounding in WT and *jar1–1* seedlings. **A**: Ten-days old seedlings were submitted to leaf wounding and 2nd leaf was harvested at the indicated time points. Total RNA was submitted to RT-qPCR with *JAR2*-specific primers. **B**: Ten-days old seedlings were submitted to 100 mM NaCl stress and 2nd leaf was harvested at the indicated time points. Total RNA was submitted to RT-qPCR to reveal *JAR2*-specific expression. Values are means and SD from 3 independent biological replicates. (DOCX 48 kb)

Additional file 5: **Figure S5.** SDS-PAGE analysis of recombinant AH8 and AH7 protein purification. AH8 and AH7 coding sequences were cloned in pHMGWA to be expressed as His-MBP-AH fusion proteins. **a**: Soluble AH8 bacterial lysate was submitted successively to amylose affinity (MBP-trap) followed by gel filtration chromatography. **b**: soluble AH7 bacterial lysate was submitted to immobilized metal-affinity chromatography (His-Trap). (DOCX 97 kb)

Additional file 6: **Table S1.** Sequences of primers for genes of interest used in the study. (DOCX 89 kb)

Additional file 7 **Table S2.** LC-MS parameters for endogenous jasmonate detection. (DOCX 38 kb)

## Data Availability

All data supporting the conclusions of this article are provided within the article and its supplementary (Additional file 1: Figure S1, Additional file 2: Figure S2, Additional file 3: Figure S3; Additional file 6: Table S1, Additional file 7: Table S2).
