# Supplementary material for: Characterization of Jasmonoyl-Isoleucine (JA-Ile) Hormonal Catabolic Pathways in Rice upon Wounding and Salt Stress
Source: Rice (N Y). 2019 Jun 25;12:45. doi: 10.1186/s12284-019-0303-0 (PMC6592992; doi:10.1186/s12284-019-0303-0)

Figure S3: *AH* gene expression in leaves upon leaf wounding

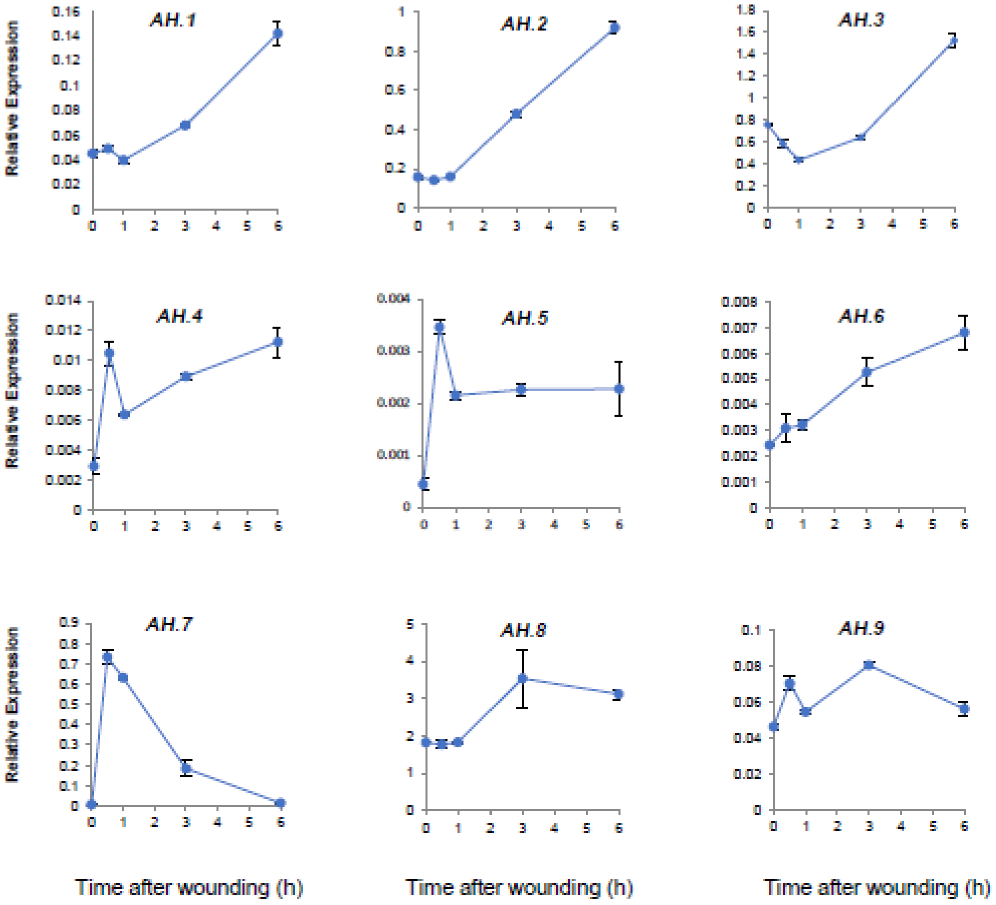

Supplement: Supplementary file 3 [file 12284_2019_303_MOESM3_ESM.docx]
