# Supplementary material for: Characterization of Jasmonoyl-Isoleucine (JA-Ile) Hormonal Catabolic Pathways in Rice upon Wounding and Salt Stress
Source: Rice (N Y). 2019 Jun 25;12:45. doi: 10.1186/s12284-019-0303-0 (PMC6592992; doi:10.1186/s12284-019-0303-0)

Figure S4: Expression profile of *OsJAR2* upon salinity stress and leaf wounding in WT and *jar1-1* seedlings.

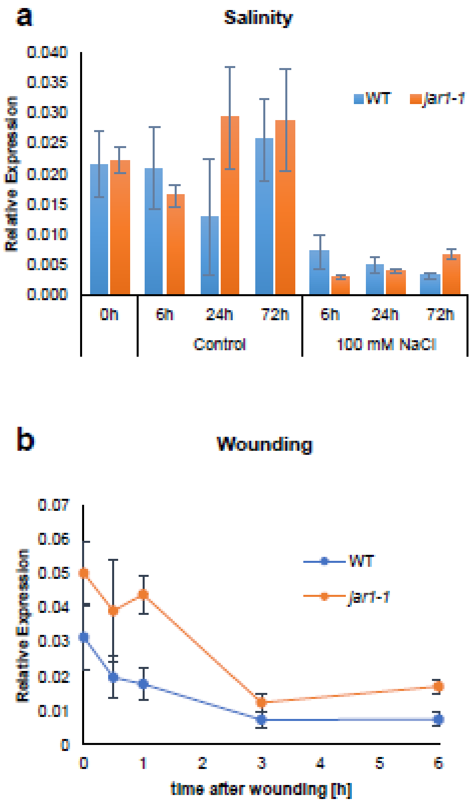

Supplement: Supplementary file 4 [file 12284_2019_303_MOESM4_ESM.docx]
