# Supplementary material for: Characterization of Jasmonoyl-Isoleucine (JA-Ile) Hormonal Catabolic Pathways in Rice upon Wounding and Salt Stress
Source: Rice (N Y). 2019 Jun 25;12:45. doi: 10.1186/s12284-019-0303-0 (PMC6592992; doi:10.1186/s12284-019-0303-0)
Supplement: Supplementary file 5 [file 12284_2019_303_MOESM5_ESM.docx]

Figure S5: SDS-PAGE analysis of recombinant AH8 and AH7 protein purification

a **His-MBP-OsAH8 purification**

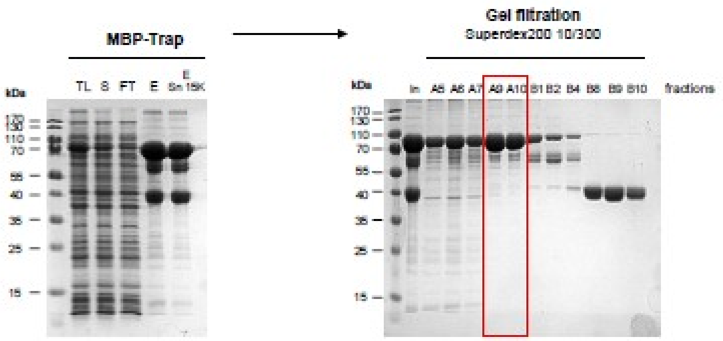

MBP, maltose-binding protein; TL, total lysate; SL, soluble lysate; FT, flow-through; E, elution; E sn 15K : 15,000 g supernatant fraction

In, input; A5 to B10 : eluted fractions

b **His-MBP-OsAH7 purification**

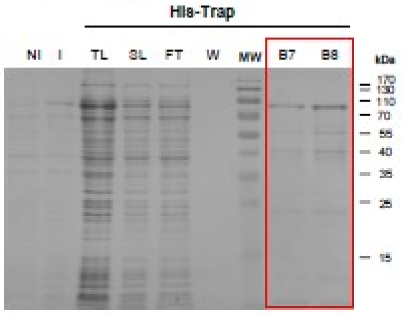

NI, non-induced; I, induced; TL, total lysate; SL, soluble lysate; FT, flow-through; W, wash; B7 and B8, eluted fractions
