# Supplementary material for: Characterization of Jasmonoyl-Isoleucine (JA-Ile) Hormonal Catabolic Pathways in Rice upon Wounding and Salt Stress
Source: Rice (N Y). 2019 Jun 25;12:45. doi: 10.1186/s12284-019-0303-0 (PMC6592992; doi:10.1186/s12284-019-0303-0)
Supplement: Supplementary file 6 [file 12284_2019_303_MOESM6_ESM.docx]

Table S1. Sequences of primers for genes of interest used in the study

qRT-PCR analysis

Gene Name	Accession Number (MSU)	Forward	Reverse
OsCYP94B4	Loc_Os11g29290	TCATGACGGCTCACATGG	ATTCCCCTCCTGTGCCATT
OsCYP94B5	Loc_Os12g25660	CTCAAGGTGACGGTGAGGAG	TTCGAATGTTAACAGCGTCA
OsCYP94C2a	Loc_Os11g05380	GGAGACTTCACTTCTACTGCAACA	ACGAGGAAACCATGACGAAC
OsCYP94C3	Loc_Os01g63930	ATGACTCCGTGACCACTCGT	GAATCTACCGCTCCATCTCG
OsCYP94C4	Loc_Os05g37250	CACATGGAAAGCTGCACTGA	CAGAGAAATCTTGAGTGGAGGA
OsCYP94D5a	Loc_Os11g04290	GATTTGCCCTGGGTTGAATTG	GCAGTAAGAACAACACCTGAAAG
OsCYP94D5b	Loc_Os12g04100	TTGCCGCTGAATGGTACTATAA	CAATACAGTGATACGCCATCCT
OsCYP94D7	Loc_Os01g59020	CTAGGCTAGCTGGACTTGTCG	GGAGCACGACACCAGTTCTA
OsCYP94D9	Loc_Os01g59000	GCCAGCTGTTCTCGACG	GCTCCCAGAAATGTGAACAATTC
OsCYP94D11	Loc_Os01g58970	TAGAGTTGCTCTTGCTTTGATCC	GGTTGTGCTAGGCCAGG
OsCYP94D12	Loc_Os01g58960	ACTACGCTGTTAGAGAGTTGC	GTAAGCTCGAAAGGTGCCG
OsCYP94D15	Loc_Os03g12260	GCCCGTAGCAGAAAGAAACA	GCCACCAACGTAGCTTTAGG
OsCYP94E2	Loc_Os01g72260	CCAATCATGTTGGGGGTAAA	GGGGCTTATTTTATGCTGGA
OsCYP94E3	Loc_Os05g31740	TGATGCCACTTGTTGTTCTTG	GAAACGGGTGAATGTCCAAC
OsAH1	Loc_Os04g44110	CTGGAGTTATGTTTGGCATTGG	TCCTCATCAACGAAGAACTTAGG
OsAH2	Loc_Os07g14590	TGGGAATTTTGTGTGTTCCA	TGCTTTGCACTGTAATTCACTC
OsAH3	Loc_Os07g14610	GAGGCAAAGAAGTATCGTTTGG	ATGCCCTATGTATGGCGAGA
OsAH4	Loc_Os07g14600	CGATCGACCTGTGGTGAAA	GCCAGCAAGCTATGACAAATAC
OsAH5	Loc_Os03g62070	TGGACAATGCTAGAAAGTCTTACA	TGGGCAGCTAGTACCATTATTT
OsAH6	Loc_Os03g62060	CCTATGCATGGCTTGGTAATG	CTGTTCTTGGGCAGCTAGTA
OsAH7	Loc_Os06g47620	ACTCCCTTAAATGTATACCGAATCT	CGCCGAGGGAGTCTATTTG
OsAH8	Loc_Os01g37960	TCTCTCCCTCTCCTTCTCTCT	GATGGGCATGCCACCTTAAT
OsAH9	Loc_Os01g51060	TTAATCTAGCGCACCGGAAG	CGCCTAGTGTGCCTTCTTTA
OsJAR2	Loc_Os01g12160	TAGTGTTGCACCTGTGACCAG	GACATCATCAACAGGAGGCCA
OsJAZ11	Loc_Os03g08320	CAGCCTTGCCTACCAGACATG	GACGATCCTGTTCTTCCTCTTCTC
OsGAPDH	Loc_Os08g03290	CTGATGATATGGACCTGAGTCTACTTTT	CAACTGCACTGGACGGCTTA
OsUBQ10	Loc_Os02g06640	GAGCCTCTGTTCGTCAAGTA	ACTCGATGGTCCATTAAACC
OsZCF61	Loc_Os07g45350	GAGAAGCTGAAGGGGGCAAT	CACACGGCACAAAAACAGCA
Osβ-ACTIN	Loc_Os10g36650	ATGCCATTCTCCGTCTT	GCTCCTGCTCGTAGTC

Recombinant Protein Expression

Gene Name	Accession Number (MSU)	Forward	Reverse
OsCYP94B5	Loc_Os12g25660	CGGGATCCCGATGGATTTGGAGAGCTGCAG	GGAATTCCTCATGTCTCGCACGTGC
OsCYP94C2a	Loc_Os11g05380	CGGGATCCCGATGGATGCCACCACCAC	GGAATTCCCTAAGTGTGAACGCGGCG
OsCYP94C4	Loc_Os05g37250	CGGGATCCCGATGGGCGCGGACGCCAC	GGAATTCCCTAACTTGGCGCGCAGTGC
OsAH7	Loc_Os06g47620	GGGGACAAGTTTGTACAAAAAAGCAGGCTT CTGCACGTTCCTGGAGGAGGACGT	GGGGACCACTTTGTACAAGAAAGCTGGGTCTCACG ACGCCTCCAGCTCGACATC
OsAH8	Loc_Os01g37960	GGGGACAAGTTTGTACAAAAAAGCAGGCTT CGGGGCGGCGCTGGACGACCC	GGGGACCACTTTGTACAAGAAAGCTGGGTCCTACAG CTCGTCATGTGCTTTCGCC
