# Supplementary material for: Characterization of Jasmonoyl-Isoleucine (JA-Ile) Hormonal Catabolic Pathways in Rice upon Wounding and Salt Stress
Source: Rice (N Y). 2019 Jun 25;12:45. doi: 10.1186/s12284-019-0303-0 (PMC6592992; doi:10.1186/s12284-019-0303-0)
Supplement: Supplementary file 7 [file 12284_2019_303_MOESM7_ESM.docx]

**Table S2**. LC-MS parameters for endogenous jasmonate detection

| Compound (ionisation mode) | Retention Time (min) | MRM transition / Detection parameters |
|---|---|---|
| 12OH-JA (-) | 6.61 | 225.2 > 59.3 [13 V] |
| 12OH-JA-Ile (-) | 7.97 | 338.3 > 130.2 [18 V] |
| 12COOH-JA-Ile (+) | 7.94 | 354.3 > 205.0 [17 V] |
| Dihydro-JA (+) | 9.03 | 213.0 > 195.1 [5 V] |
| Dihydro-JA-Ile (+) | 9.62 | 326.2 > 128.2 [14 V] |
| JA-Ile (-) | 9.36 | 322.3 > 130.2 [18 V] |
| OPDA (+) | 10.35 | 293.3 > 275.3 [13 V] |
| JA (-) | 8.57 | 209.3 > 59.3 [10 V] |
| JA-Cys (+) | 8 | 314.2 > 151.2 [11 V] |
| JA-Ile (-) | 9.36 | 322.3 > 130.2 [18 V] |
| JA-Glu (-) | 7.75 | 338.3 > 128.2 [15 V] |
| JA-Gly (+) | 7.82 | 268.3 > 151.1 [9 V] |
| JA-Phe (+) | 9.37 | 358.3 > 120.1 [23 V] |
| JA-Phe (-) | 9.37 | 356.3 > 164.1 [16 V] |
| JA-Pro (+) | 8.49 | 308.3 > 151.1 [11 V] |
| JA-Pro (-) | 8.49 | 306.3 > 114.2 [16 V] |
| JA-Trp (+) | 9.12 | 397.3 > 188.0 [16 V] |
| JA-Tyr (+) | 8.29 | 374.3 > 328.1 [10 V] |
| JA-Tyr (-) | 8.3 | 372.3 > 180.1 [17 V] |
